# Pulmonary Nodule Recognition Based on Multiple Kernel Learning Support Vector Machine-PSO

**DOI:** 10.1155/2018/1461470

**Published:** 2018-04-29

**Authors:** Yang Li, Zhichuan Zhu, Alin Hou, Qingdong Zhao, Liwei Liu, Lijuan Zhang

**Affiliations:** ^1^School of Mathematics and Statistics, Northeast Normal University, Changchun, Jilin 130024, China; ^2^School of Computer Science and Engineering, Changchun University of Technology, Jilin 130012, China; ^3^Faculty of Statistics, Jilin University of Finance and Economics, Changchun, Jilin 130117, China

## Abstract

Pulmonary nodule recognition is the core module of lung CAD. The Support Vector Machine (SVM) algorithm has been widely used in pulmonary nodule recognition, and the algorithm of Multiple Kernel Learning Support Vector Machine (MKL-SVM) has achieved good results therein. Based on grid search, however, the MKL-SVM algorithm needs long optimization time in course of parameter optimization; also its identification accuracy depends on the fineness of grid. In the paper, swarm intelligence is introduced and the Particle Swarm Optimization (PSO) is combined with MKL-SVM algorithm to be MKL-SVM-PSO algorithm so as to realize global optimization of parameters rapidly. In order to obtain the global optimal solution, different inertia weights such as constant inertia weight, linear inertia weight, and nonlinear inertia weight are applied to pulmonary nodules recognition. The experimental results show that the model training time of the proposed MKL-SVM-PSO algorithm is only 1/7 of the training time of the MKL-SVM grid search algorithm, achieving better recognition effect. Moreover, Euclidean norm of normalized error vector is proposed to measure the proximity between the average fitness curve and the optimal fitness curve after convergence. Through statistical analysis of the average of 20 times operation results with different inertial weights, it can be seen that the dynamic inertial weight is superior to the constant inertia weight in the MKL-SVM-PSO algorithm. In the dynamic inertial weight algorithm, the parameter optimization time of nonlinear inertia weight is shorter; the average fitness value after convergence is much closer to the optimal fitness value, which is better than the linear inertial weight. Besides, a better nonlinear inertial weight is verified.

## 1. Introduction

The number of deaths from lung cancer is as high as 137 million annually around the world, accounting for 18% of cancer mortality ratio. Early surgical treatment is the most effective treatment for lung cancer, but most patients are diagnosed in the late stage of the disease. In 2015, the European Academy of Imaging and the European Respiratory Society published the latest white paper on lung cancer screening in European Respiratory Journal (ERJ) to guide clinical lung cancer screening for early detection and early treatment of lung cancer.

As early representation form of lung cancer in the lung CT image, a pulmonary nodule is defined as a nearly spherical opacity with a diameter smaller than 3 cm. Computed Tomography (CT) technology is an important means of early detection of pulmonary nodules disease. According to the CT characterization, pulmonary nodules can be divided into solid nodules (such as solitary pulmonary nodules, pulmonary wall adhesion nodules, and vascular adhesion nodules), ground glass nodules, and cavitary nodules.

Computer-Aided Detection (CAD) system of lung is one of the applications of machine vision; it can reduce overload visual fatigue of the radiologist and decrease the possibility of the resulting miscarriage or omission and also provide auxiliary diagnosis results for the doctor as “third party.” Usually, the lung CAD system includes the following modules: acquisition of the lung CT image data, preprocessing of CT image, lung parenchyma segmentation, detection of VOI (Volume of Interest) or ROI (Region of Interest) in candidate nodules images (mainly refers to the extraction or segmentation), calculation and selection of ROI or VOI features, and recognition of pulmonary nodules, where pulmonary nodules recognition is the core module of the CAD system. The algorithm of Support Vector Machine (SVM) has been widely used in the detection and recognition of pulmonary nodules (see, e.g., [1–15]). Among them, Li et al. [1] applied mixed kernel SVM algorithm to distinguish benign and malignant lung nodules, making the recognition accuracy (ACC) reach 92% and the sensitivity index reach 92.59%; Wang et al. [2] detected lung lesions by use of three-dimensional SVM with Latent Variable algorithm. Furthermore, Demir and Çamurcu [16] and Chang et al. [17] imported the algorithm of Particle Swarm Optimization (PSO) into SVM and selected the optimal parameter group by PSO and then used SVM for identification. In addition, Ma et al. [18] adopted the method of multiple classifiers fusion for lung disease identification.

The Multiple Kernel Learning Support Vector Machine (MKL-SVM) algorithm has achieved good recognition accuracy not just in recognition of lung nodules in [1] but also in other application fields (see [19, 20]). In [21], the Multiple Kernel Learning (MKL) method was elaborated and the latest research progresses were presented in this field. However, the MKL-SVM algorithm involves a large number of parameters, and the selection of parameters will have an important impact on the actual results. In [1], the selection of the optimal parameters is obtained by the grid search algorithm. The advantage of the grid search algorithm is the easiness to get the global optimal solution in the case of dense mesh division, but the disadvantage of the method is that it has a large amount of computation, a long time to search, and a poor real-time performance, which is not easy to form online identification algorithm. The selection of the relevant parameters is an urgent problem to be solved in the MKL-SVM, and the Particle Swarm Optimization (PSO) algorithm based on swarm intelligence algorithm provides an idea to solve the problem.

In this paper, the PSO algorithm and MKL-SVM algorithm are combined to realize the parameter optimization of the MKL-SVM. On this basis, the PSO algorithm with different inertia weights was compared and analyzed in order to obtain the parametric array similar or superior to that of the grid search algorithm aiming at quickly searching the optimal parametric array and the reasonable inertia weight and then precise identification of the pulmonary nodules.

## 2. Multiple Kernel Learning Support Vector Machine (MKL-SVM)

### 2.1. Support Vector Machine

SVM is a learning method using small amount of samples, which can be applied to predict or classify unknown samples by structural risk minimization. The training sample is represented as follows:

(1) $$\begin{array}{c} T=\left\{\;\left(\;x_{i},y_{i}\;\right)\;\right\},\;i=1,2,\ldots ,l, \end{array}$$

where* l* is the number of training samples, *x*_*i*_ denotes the input vector of SVM, corresponding to the feature of the above *N*-dimension region of interest (ROI), *x*_*i*_ ∈ *R*^*N*^;  *y*_*i*_ ∈ {−1, +1} indicates category label; here, *y*_*i*_ = 1 corresponds to nodules and *y*_*i*_ = −1 corresponds to nonnodules.

When SVM is used in the two classification problems, the original model can be written as the following nonlinear optimization problem:

(2) minw,b,ξ 12w2+C∑i=1lξis.t. yiw•Φxi+b≥1−ξi,i=1,2,…,lξi≥0,  i=1,2,…,l,

where *w* is the weight vector and *b* is the threshold, and the aim of SVM is to maximize the classification interval 2/‖*w*‖, that is, minimization of ‖*w*‖^2^.* C* is the regularization coefficient or penalty parameter, which describes the degree of penalty for misclassification samples. The greater *C* is, the more obvious the penalty for misclassification is. When the data cannot be completely separated, the maximum interval will be negative, thus introducing slack variables *ξ*_*i*_ which can measure the distance between the actual output *y*_*i*_ and the Support Vector Machine output.

In the feature space, SVM is used to map the input data (*X*_*i*_) into a high-dimensional feature space *Z* by nonlinear transformation Φ(*X*), and then the optimal classification hyperplane is constructed in the high-dimensional feature space *Z* to realize the SVM. In the process of constructing the hyperplane in the feature space *Z*, the training algorithm uses the dot product and the kernel function *K*(*x*_*i*_, *x*_*j*_) to represent the inner products Φ(*x*_*i*_) and Φ(*x*_*j*_); that is, a function *K* can be found to form the next formula:

(3) $$\begin{array}{c} K\left(\;x_{i},x_{j}\;\right)=\Phi \left(\;x_{i}\;\right)\cdot \Phi \left(\;x_{j}\;\right). \end{array}$$

Thus, it is not necessary to construct and solve the convex quadratic programming problem for a given training sample, and the problem is transformed into the following optimization problem by using Lagrange multiplier:

(4) minα 12∑i−1l ∑j=1lyiyjKxi,xjαiαj−∑j=1lαjs.t. ∑i=1lyiαi=0,0≤αi≤C,  i=1,2,…,l.

The offset *b* in (2) can be solved by means of the following equation:

(5) $$\begin{array}{c} b=y_{j}-\overset{l}{\underset{i=1}{\sum }}y_{i}\alpha_{i}K\left(\;x_{i},x_{j}\;\right). \end{array}$$

The decision function is constructed as follows:

(6) $$\begin{array}{c} f\left(\;x\;\right)=sgn\left(\;g\left(\;x\;\right)\;\right), \end{array}$$

where

(7) $$\begin{array}{c} g\left(\;x\;\right)=\overset{l}{\underset{i=1}{\sum }}\alpha_{i}y_{i}K\left(\;x_{i},x_{j}\;\right)+b. \end{array}$$

### 2.2. Multiple Kernel Learning SVM (MKL-SVM)

Various kernel functions have diverse advantages. One of the keys to improve the performance of SVM is to design an appropriate kernel function for a given problem. The common basic kernel functions are polynomial kernel function and radial basis function (RBF), which are presented, respectively, as follows:

(8) Kpolyx,y=xty+1dKrbfx,y=exp⁡−x−y22g2,

where the parameter *d* represents the order of the polynomial kernel, the parameter *g* denotes the width of the RBF kernel, and* d* and *g* need to be given in advance.

**Proposition 1 .**

The convex combination form of the kernel function is still a kernel function:

(9) $$\begin{array}{c} K_{mix}\left(\;x,x^{\prime }\;\right)=\overset{U}{\underset{p=1}{\sum }}m_{p}K_{p}\left(\;x,x^{\prime }\;\right), \end{array}$$

where

(10) $$\begin{array}{c} \overset{U}{\underset{p=1}{\sum }}m_{p}=1,\;0<m_{p}<0,\;\;p=1,\ldots ,U, \end{array}$$

and *K*_*p*_ is the* p*th species of basic kernel function and *m*_*p*_ corresponds to the weights of the* p*th species of basic kernel function in the total multiple kernel function.* U* species basic kernel functions are used in the multiple kernel function, and the weight sum of various basic kernel functions is one so as to limit the weight proportion of various basic kernel functions in the multiple kernel functions in proportion.

**Proof**

Let {*x*_1_, *x*_2_,…, *x*_*l*_} be a set of *l* points in any given *R*^*N*^; we just need to prove that the Gram matrix in (9) is positive semidefinite matrix.

Let *K*_1_, *K*_2_,…, *K*_*P*_ be the Gram matrix of *K*_1_(*x*, *x*′), *K*_2_(*x*, *x*′),…, *K*_*P*_(*x*, *x*′) for {*x*_1_, *x*_2_,…, *x*_*l*_}; for any *α* ∈ *R*^*l*^, we obtain

(11) αTKmixx,x′α=αT∑p=1UmpKpx,x′α=αTm1K1x,x′+m2K2x,x′+⋯+mUKUx,x′α=m1αTK1x,x′α+m2αTK2x,x′α+⋯+mUαTKUx,x′α≥0.

So *K*_mix_(*x*, *x*′) = ∑_*p*=1_^*U*^*m*_*p*_*K*_*p*_(*x*, *x*′) is positive semidefinite matrix; that is, *K*_mix_(*x*, *x*′) is a kernel function, and the evidence is proven.

It is proven that the kernel function expressed by (9) satisfies the Mercer condition and can be used for the training and classification of SVM. By using the above MKL-SVM, we can use nonlinear transformation of the sample points to get the corresponding kernel matrix so as to obtain the classification results during the training of SVM classifier.

RBF kernel has a strong ability to learn, and polynomial kernel has strong generalization ability; thus the combination of the two can take into account the ability of both learning and generalization. If we use only two kinds of basic kernel functions of both RBF kernel and polynomial kernel, that is, *U* = 2, *K*_1_ = *K*_poly_, and *K*_2_ = *K*_rbf_, the multiple kernel function of (12) is able to be formed. Compared with the single kernel function, we need to estimate a set of kernel parameters and a weight coefficient *m*. The weight coefficient *m* can regulate freely the proportion of different kernel functions mixed in multiple kernels, adjust flexibly the ability of learning and generalization, and make the results unbiased towards the promotion of a particular target.

(12) $$\begin{array}{c} K\left(\;x,x^{\prime }\;\right)=mK_{poly}\left(\;x,x^{\prime }\;\right)+\left(\;1-m\;\right)K_{rbf}\left(\;x,x^{\prime }\;\right). \end{array}$$

In [1], the grid search algorithm in the sense of CV is used to find the optimal regularization coefficient* C*, the order *d* of the polynomial kernel, the kernel width *g* of the RBF kernel, and the weight coefficient *m* of the multiple kernels. The optimal parameter group can be obtained by the grid search algorithm during the CV process corresponding to the highest classification accuracy. Lots of parameters and short step length of mesh induce large amount of calculation and long running time. The global optimal solution could be found by the heuristic algorithm, not needing to traverse all the parameter points in the grid.

### 2.3. MKL-SVM Based on Modified Particle Swarm Optimization Algorithm

Particle Swarm Optimization (PSO) is a typical heuristic algorithm on the basis of swarm intelligence optimization theory. In 1955, PSO was first proposed by Kennedy and Eberhart in [22], whose basic principle was originated from the research on the predation behavior of artificial life and birds. When birds prey upon food, the simplest and most effective method of finding food is to search the current area around the food nearest to birds. Compared with Generic Algorithm (GA), PSO searches the optimal particles by tracking the particles in the solution space without selection, crossover, and mutation.

It is assumed that the population *X* = (*X*_1_, *X*_2_,…, *X*_*n*_) consists of *n* particles in a *D*-dimensional search space, where *X*_*i*_ represents the position of the *i*th particle in *D*-dimensional search space and also is a candidate solution of problem denoted by a vector of *D* dimensions as *X*_*i*_ = (*X*_*i*1_, *X*_*i*2_,…,*X*_*iD*_)^*T*^. According to the objective function, we can calculate the fitness value of each particle position *X*_*i*_. The speed of the *i*th particle is *V*_*i*_ = (*V*_*i*1_, *V*_*i*2_,…,*V*_*iD*_)^*T*^, and its individual extreme value and group extreme value are *P*_*i*_ = (*P*_*i*1_, *P*_*i*2_,…,*P*_*iD*_)^*T*^ and *P*_*g*_ = (*P*_*g*1_, *P*_*g*2_,…,*P*_*gD*_)^*T*^, respectively. During each iteration, the particle updates its velocity and position by the individual extrema and the group extrema, which are given, respectively, as follows:

(13) Vidk+1=ωVidk+c1r1Pidk−Xidk+c2r2Pgdk−Xidk,Xidk+1=Xidk+Vidk+1,

where *ω* is an inertia weight; *d* = 1,2,…, *D*; *D* represents the number of parameters to be searched; *k* is the number of the present iterations; *V*_*id*_ is the velocity of particles, *c*_1_ and *c*_2_ are acceleration factors, which are nonnegative constants, and *r*_1_ and *r*_2_ are random numbers distributed within the interval [0,1]. In order to prevent the blind search of particles, the position and velocity are usually limited to the range of [−*X*_max_, *X*_max_] and [−*V*_max_, *V*_max_].

The PSO algorithm is applied into MKL-SVM algorithm of (12). Because the corresponding order of polynomial kernel is defined as positive integer for *d*⩾2 and with the increase of *D*, generalization ability of polynomial kernel decreases gradually, so only the two values *d* = 2 and *d* = 3 were calculated, and there is no need to search other parameters. Here the dimension of the search space of the particle is set to *D* = 3; *X*_*i*_ = (*X*_*i*1_, *X*_*i*2_, *X*_*i*3_)^*T*^ represents the solution of the *i*th particles, where *X*_*i*1_, *X*_*i*2_, and *X*_*i*3_ of each dimension are corresponding to the regularization coefficient* C*, the kernel width *g* of RBF, and the multiple kernel weight *m* to be searched, respectively.

## 3. Application of MKL-SVM-PSO Algorithm in Pulmonary Nodule Recognition

After introducing the classic PSO algorithm, the recognition accuracy rate (ACC) of pulmonary nodules in the sense of CV is regarded as the final target and determined as the fitness function value of PSO, and then ACC is defined as follows:

(14) $$\begin{array}{c} ACC=\frac{\left(TP+TN\right)}{TP+TN+FP+FN}, \end{array}$$

where TP denotes the detected true positive nodule; FP is the detected false positive nodule; FN indicates the undetected false negative nodule; TN is the detected true negative nodule, that is, nonnodule. ACC measures total recognition accuracy to measure the actual detection rate of pulmonary nodules; the SEN is defined as follows:

(15) $$\begin{array}{c} SEN=\frac{TP}{\left(TP+FN\right)}. \end{array}$$

**Figure 1:**
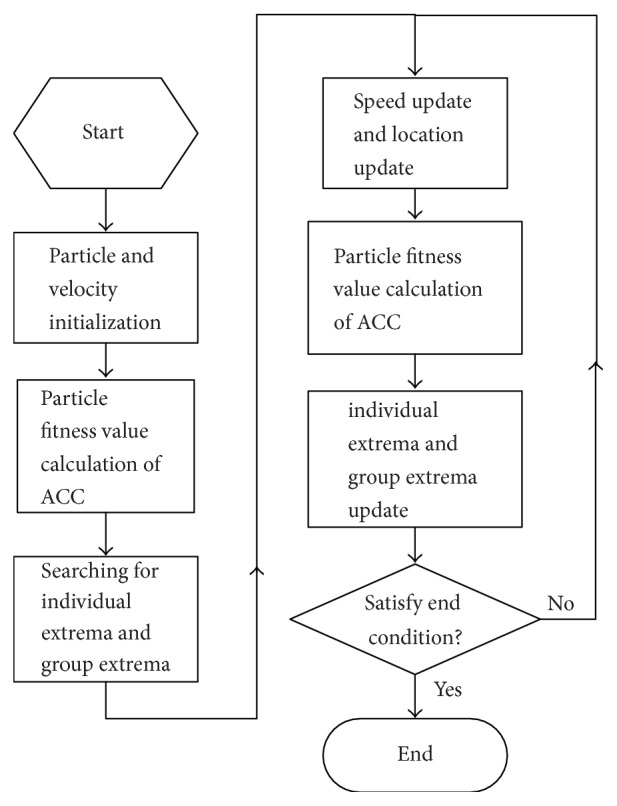
The diagram of MKL-SVM-PSO algorithm.

The parameter optimization algorithm of MKL-SVM-PSO algorithm is described in Figure 1.

## 4. Experimental Results and Analysis

### 4.1. The Experimental Data and the Results Analysis of MKL-SVM-PSO Algorithm

The experimental data were collected from 20 groups from a third-grade class A hospital in Jilin province with a total of about 700 images, and each group was diagnosed with the diagnostic criteria of doctor. The size of each CT image was 512 × 512, and the slice thickness is 5.0 mm. 270 ROI are extracted: 80 pulmonary nodules and 190 false positive nodules. After feature selection, the data samples were randomly divided into two groups: the training group including 170 samples and the testing group including 100 samples.

**Figure 2:**
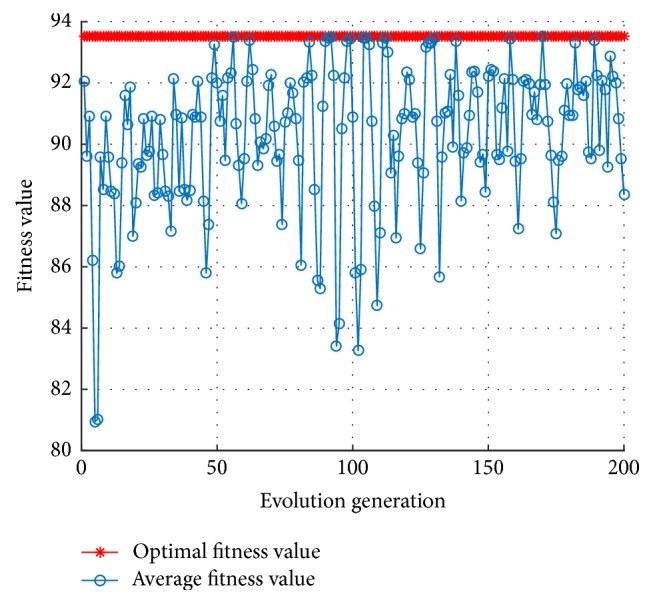
The fitness curve of the MKL-SVM-PSO algorithm with constant inertia weight.

The simulation experiments are carried out using the platform MATLAB with libsvm toolbox. In the process of model parameter optimization, 5-fold cross-validation is used to obtain the optimal parameter set corresponding to the highest ACC. Let the number *n* of cluster particles be 20, that is, *n* = 20 in the MKL-SVM-PSO algorithm, and the dimension *D* of each particle be 3, that is,* D* =3, and then the maximum number of iterations maxgen of the algorithm is set to be 200, and the inertia weight is constant, that is, *ω* = 1. The simulation experiment will show the convergence performance through the optimal fitness curve and the average fitness curve as in Figure 2.

The optimal individual fitness curve of MKL-SVM-PSO algorithm is obtained as shown in Figure 2. The most optimal individual fitness value, namely, the nodule recognition accuracy, is 94.1176% through 5-fold cross-validation in training set; and the correspondent optimal particle position is *C* = 29.7267, *g* = 19.0653, and *d* = 2, *m* = 0.8, respectively. In this case, the running time of the proposed MKL-SVM-PSO algorithm is 363.4640 s, less than 2815.0786 s compared with 3178.5426 s of the grid search algorithm [1], and it accounts for just 11.43% of the grid search algorithm running time. Applying the proposed method to the test set, the test results of ACC reached 91% and those of SEN reached 88.89%.

### 4.2. The Influence and Comparison of Different Inertia Weight on Lung Nodule Recognition

Compared with the grid search algorithm, the computational time of MKL-SVM-PSO algorithm requires shorter time, but as the iteration times gradually increase, the shock amplitude of average fitness value in each generation is more severe, and a certain gap exists with the optimal fitness value, which can be found in Figure 2.

**Figure 3:**
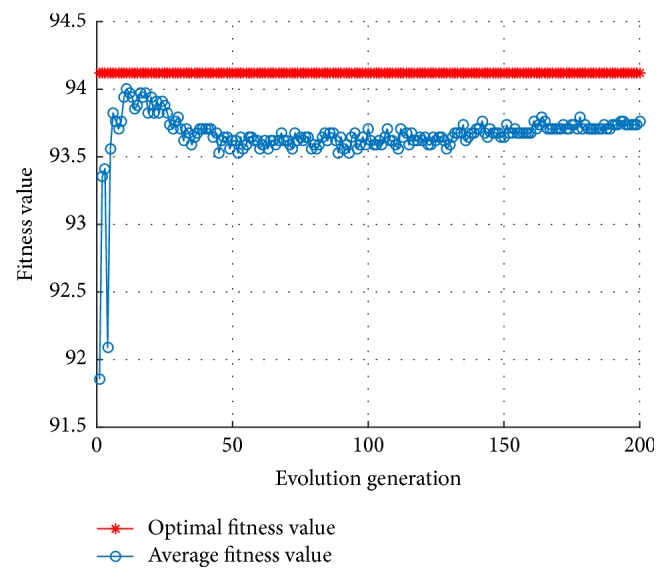
The fitness curve of MKL-SVM-PSO algorithm of (16).

In [23], the inertia weight *ω* was first introduced into PSO algorithm, and the larger inertia weight value that was conducive to the global search was pointed out, and the smaller inertia weight that was more conducive to the local search was also presented, and, moreover, the inertia weight that can reflect the ability of the particle to inherit the previous speed was also discussed. In order to balance the global search ability and local search ability of the algorithm, the linear decreasing inertia weight (LDIW) method proposed by Shi and Eberhart [23] will be used to reassign *ω* as follows:

(16) $$\begin{array}{c} \omega \left(\;k\;\right)=\frac{\omega_{start}\left(\omega_{start}-\omega_{end}\right)\left(T_{max}-k\right)}{T_{max}}, \end{array}$$

where *ω*_start_ is the initial inertia weight; *ω*_end_ is the inertia weight of the iteration to the maximum number of times; *k* is the number of the current iteration generations; *T*_max_ is the maximal iteration number. In order to ensure that the above iteration algorithm not only has better global search ability in initial phase but also has strong local search ability to obtain the optimal solution in later iterations, commonly let *ω*_start_ = 0.9 and *ω*_end_ = 0.4; the inertia weight decreases linearly from initial 0.9 to 0.4, and this is also an empirical approach. The fitness curve of the optimal parameters obtained by the MKL-SVM-PSO algorithm is shown in Figure 3.

It can be seen from Figure 3 that the shock amplitude is reduced by using the inertia weight of (16) rather than using constant inertia weight, close to the optimal solution in the early stage. Besides, commonly used linear inertia weight is shown in the following equation:

(17) $$\begin{array}{c} \omega \left(\;k\;\right)=\omega_{end}+\left(\;\omega_{start}-\omega_{end}\;\right)\left(\;\frac{T_{max}-k}{T_{max}}\;\right). \end{array}$$

**Figure 4:**
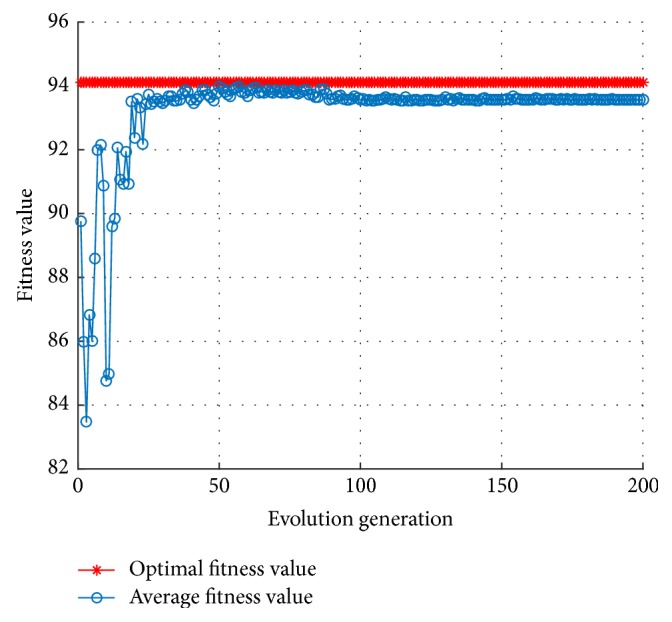
The fitness curve using the MKL-SVM-PSO algorithm of (17).

Figure 4 shows the fitness curve corresponding to (17). In Figure 4, the shock amplitude induced by inertia weight of (17) is slightly larger, but it quickly converges to the optimal individual fitness value. The linear inertia weight represented by (16) and (17) can make the average fitness curve smooth, though it is easy to fall into local optimum in the early stage.

In order to ensure obtaining the global optimal solution, the following three kinds of nonlinear inertia weight are adopted to control convergence precision and convergence speed, so that the average fitness values reach the best fitness value index quickly and smoothly.

(18) ωk=ωstart−ωstart−ωendkTmax2

(19) ωk=ωstart−ωstart−ωend2kTmax−kTmax2

(20) ωk=ωendωstartωend1/1+ck/Tmax.

**Figure 5:**
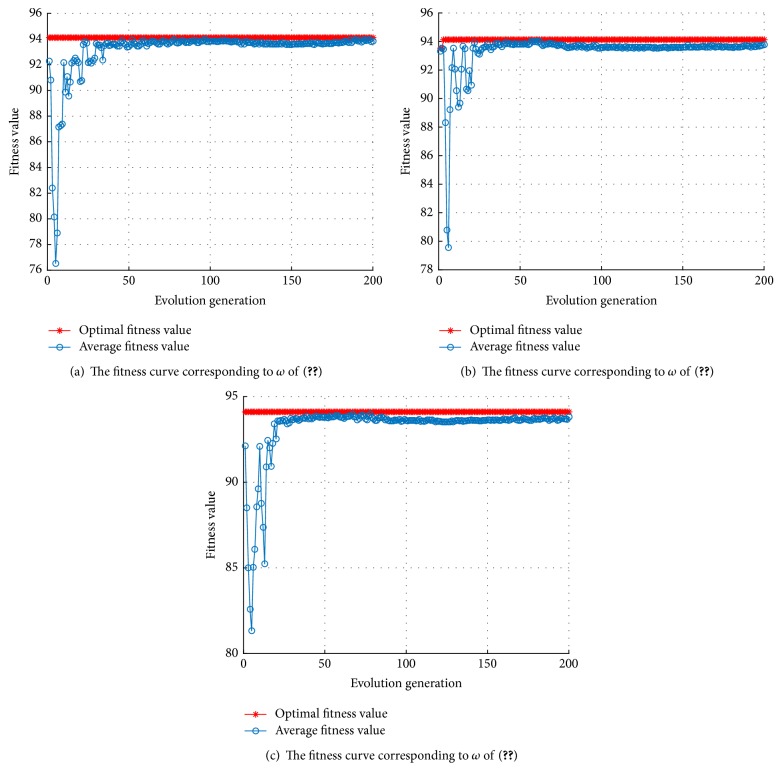
The fitness curve of optimal parameters group searching by MKL-SVM-PSO algorithm.

Using the nonlinear parameter of the above three methods, respectively, the fitness function curve represents total recognition accuracy of pulmonary nodules as shown in Figure 5.

**Figure 6:**
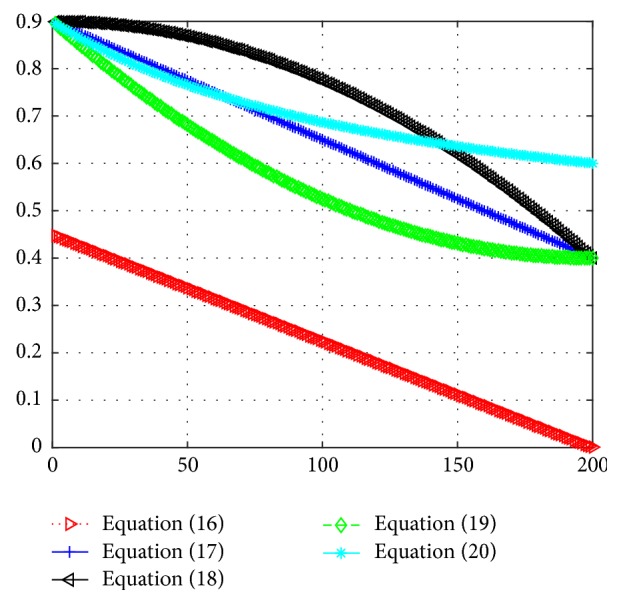
The variation curves of several dynamic inertia weights.

In order to compare the influence of different kinds of inertia weight on the parameter optimization, Figure 6 describes the curve of five kinds of dynamic weight corresponding to (16)–(20) changing along with the number of iterations. In the early stage of iteration, the larger inertia weight can make the algorithm maintain a strong global search ability, and the small inertia weight can make the algorithm search precisely in the late stage of iteration. As we know from the variation curve of several dynamic weights, the dynamic weight of (18) changes slowly in the early stage, and the value is larger so as to maintain the global search ability of the proposed algorithm; moreover, the dynamic weights change rapidly in the late stage and improve greatly the local searching ability of the algorithm; furthermore, the parameter optimization has also got a good result with the corresponding fitness curve, which is the optimal way of dynamic weights.

In summary, the Particle Swarm Optimization algorithm with constant weight has a fast convergence speed, but in the later stages it is easy to fall into local optimal solution with little accuracy. The linear inertia weights of (16) and (17) are easy to fall into local optimum. If we adopt several dynamic nonlinear inertia weight methods from (18) to (20), the algorithm converges slowly in initial stage, but in the later period local search ability is enhanced, which makes the algorithm jump out of local optimum and get the global optimal solution, so as to improve the accuracy of the algorithm. The form in (18) is the optimal nonlinear inertia weight.

In order to compare several kinds of Particle Swarm Optimization algorithms with different inertial weights and the parameters optimization time and recognition results of grid search algorithm, each algorithm is operated 20 times, and the average results of 20 times are listed in Table 1.

From the experimental results in Table 1, it can be seen that the parameter optimization time of the MKL-SVM grid search algorithm is the longest, which is almost 7 times of that of the MKL-SVM-PSO algorithm, and the running speed is much slower than that of the MKL-SVM-PSO grid search algorithm. In the MKL-SVM-PSO algorithm, the inertia weight is, respectively, set to a constant value, a linear dynamic weight, and a nonlinear dynamic weight, and the parameter optimization time has a little difference as well. When the inertia weight is set to a constant value, the average running time is the shortest, and the optimum fitness is 94.1176% in the training stage, but its generalization ability is not as good as the experimental result obtained by dynamically searching the inertia weight. The average ACC value obtained from the test set is 90.45% and the average SEN is only 86.85%. The optimal fitness value is always 94.1176% during each operation process in the training stage using dynamic inertia weight from (16) to (20). The same results of every test are obtained on the test set. The ACC value is 91% and the SEN is 88.89%; only the training times are different. Among them, the average running time of (18) is the shortest, that is, 416.0204 s, which is the best among all the algorithms. The nonlinear inertial weight algorithm is faster than the linear inertial weight algorithm, which should be due to the fast convergence of the dynamic inertial weight algorithm.

**Figure 7:**
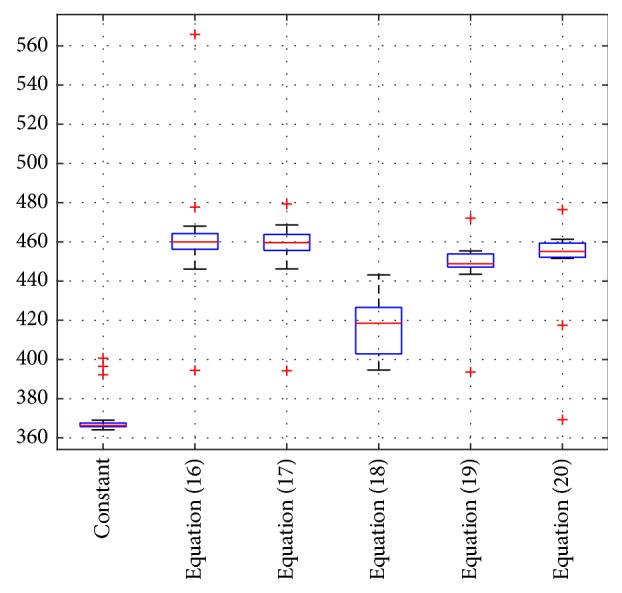
The statistical boxplot of parameter optimization time.

Figure 7 shows the 20 times statistical results corresponding to parameter optimization times. The statistical values of the boxplot are listed in Table 2.

From the data in Figure 7 and Table 2, it can be seen that when the inertia weight is constant, the box is located at the bottom, and the three outliers are also very low, and also the training time is the shortest. Combined with Figure 2, the convergence rate of the algorithm is much slow, and no obvious generation number of convergence can be found. From the test results of test set in Table 1, the generalization ability is not as good as that of the dynamic inertial weight algorithm, so it is not the optimal one. When dynamic inertial weight algorithm is adopted, the boxplot of the model training time corresponding to the three nonlinear inertial weight algorithms is located under the boxplot corresponding to the linear inertial weight. It means that the training time (i.e., parameter optimization time) of nonlinear inertial weight is less than that of linear inertial weight, and the parameter optimization time obtained by (18) is the shortest and there are no outliers in it. The upper adjacent was 443.129 s, the lower adjacent was 394.638 s, and the median value was 418.49 s, respectively. The lower adjacent optimization time of the inertia weight obtained by (19) is 443.456 s, which is close to the upper adjacent value of the box obtained by (18). Furthermore, the training time of the former method is second only to that of (18). The position of box obtained by (20) is superior to that of linear inertial weight, but it is not good at nonlinear weight. The positions of the two boxes obtained by the data of both (16) and (17) are very close. The upper adjacent and lower adjacent of the box corresponding to (17) are all higher than those of (16), but the median value of (17) box is lower than that of (16) box. Therefore, the parameter optimization time of (18) is optimal.

Since the value of each inertial weight is different, the maximum number of convergence generations is different in various algorithms. Therefore, it is not reasonable to compare the Euclidean norm error between the optimal fitness value and the average fitness value after convergence, because it is difficult to express the merits and demerits of each algorithm. In order to reasonably express the Euclidean distance between the optimal fitness curve and the average fitness curve after convergence, we define the normalized Euclidean norm error as follows:

(21) Fgy=Vbestt:max⁡gen−VAveraget:max⁡gen2=Vbestt−VAvet2+Vbestt+1−VAvet+12+⋯+Vbestmax⁡gen−VAvemax⁡gen2max⁡gen−t+1,

where the average fitness curve converges from the* t*th generation. *V*_best_ and *V*_ave_ are both maxgen dimensional vectors and represent the optimal and average fitness value, respectively. Here the normalized Euclidean norm error is defined as *F*_*gy*_ and used to express the Euclidean distance between the average fitness value and the optimal fitness value of each generation after convergence.

The average values of the indexes obtained after 20 operation times are shown in Table 3. We compared and analyzed several key parameters such as the convergence generation number of the average fitness curve, the normalized Euclidean distance between the average fitness value and the optimal fitness value after reaching the convergence generation number, the median value, the maximum, the mean value, and total Euclidean norm of error vector with 200 generations in the average fitness curve. Each index is the statistical average of 20 operation results.

From above experimental results, it can be seen that when the inertia weight is constant, it is very difficult to find which generation of curves converges obviously in Table 3, so it is impossible to calculate the Euclidean norm of normalized error vector after reaching maximum number of convergence generations. The median and mean value of the average fitness curve are also the lowest among all the algorithms, which further indicates that the effect of the constant inertia weight is not as good as that of dynamic inertia weight. In the dynamic inertial weight algorithm, the maximum, mean, and median values of the average fitness curve are the highest in all the algorithms, and the Euclidean distance between the mean fitness curve and the optimal fitness curve is the smallest; that is, the least squares norm of the error vector is the smallest. The convergence is earlier, and the normalized Euclidean norm of the error vector is the smallest. However, there exists the particle premature phenomenon, so it is easy to fall into the local optimal solution. It is further proven that the global search performance in the early stage is limited. The mean value of average fitness corresponding to nonlinear inertial weight of (18) is 93.2050%, and the maximum is 94.0290%; the median value is 93.6719%. The mean value is low, and also the convergence generation number is large, and the Euclidean norm of global error vector is large because of the sharp oscillation in the initial iteration. It also reflects that the global search ability of the algorithm in the initial iteration prevents fully the particle premature convergence. Thus, the three indexes, the maximum, the median value, and the Euclidean norm of the normalized error vector after reaching convergence, are able to reflect better convergence performance after convergence. The indexes obtained by (19) and (20) are basically superior to that of (17); that is, the nonlinear inertial weight algorithm is superior to the linear inertial weight algorithm generally.

In summary, the Particle Swarm Optimization algorithm with dynamic inertia weight is better than the one with constant inertia weight, and the algorithm using nonlinear inertia weight is better than that one using linear inertia weight. The MKL-SVM-PSO algorithm has gained good results by use of dynamic nonlinear inertial weight of (18) in this paper. The algorithm has the ability of global searching at the beginning of iteration. After reaching the convergence generation number, the average fitness value can approach the optimal fitness value more smoothly and quickly, which makes it easier to find the global optimal solution.

## 5. Conclusion

In this paper, a MKL-SVM-PSO algorithm with nonlinear inertial weight is proposed to search the optimal parameter set of hybrid kernel Support Vector Machine quickly and accurately and achieved better effects in pulmonary nodule recognition. The main innovative work goes as follows:

1. The PSO algorithm is introduced into the mixture Kernels SVM algorithm and used for the discrimination of benign and malignant pulmonary nodules.
2. On the basis of changing dynamic weights, the similarities and differences between linear weights and nonlinear weights are discussed, and the optimal dynamic nonlinear weights are obtained. The average fitness value of the algorithm is close to the optimal fitness value quickly and smoothly, so that the global optimal solution is easy to be obtained.
3. The Euclidean norm index of normalized error vector is proposed to measure the difference between the optimal fitness curve and the average fitness curve after convergence with different inertial weights. The index solves the problem that different convergence generations of different algorithms result in different dimensions of error vectors in various algorithms, and it is difficult to compare the discrepancy. The validity of dynamic inertial weight algorithm is verified from the point of view of statistics.

The experimental results show that the model training time of MKL-SVM-PSO algorithm is only 1/7 of the training time of MKL-SVM grid search algorithm with better recognition effect. It can be seen that the dynamic inertia weight is better than constant inertia weight in the MKL-SVM-PSO algorithm from Table 3. Compared with the linear inertial weight algorithm, the parameter optimization speed of nonlinear inertial weight algorithm is rapid, and the average fitness value after convergence is much closer to the optimal fitness value. The dynamic inertial weight corresponding to (18) is the optimal method in this paper.

Although ACC, as a fitness value, has obtained good experimental results in this method, medical attention is often paid to the SEN index to prevent missed detection. Our next job is to extend the proposed MKL-SVM-PSO algorithm to multitarget search in order to achieve accurate identification and nonmissed detection of pulmonary nodules.

## Figures and Tables

**Table 1:** Comparison of various indexes in parameter optimization stage of different algorithms.

	Different inertial weight algorithm	Average parameter optimization time (s)	Average optimal fitness value	Average ACC value obtained from test set	Average SEN value obtained from test set
1	The constant is 1	370.7950	94.1176%	90.45%	86.85%
2	(16)	462.4134	94.1176%	91%	88.89%
3	(17)	457.0022	94.1176%	91%	88.89%
4	(18)	416.0204	94.1176%	91%	88.89%
5	(19)	448.1536	94.1176%	91%	88.89%
6	(20)	450.4456	94.1176%	91%	88.89%
7	Grid search algorithm	3096.1427	94.1176%	91%	88.89%

**Table 2:** Statistical indexes corresponding to Figure 7.

	Different inertial weight algorithm	Upper adjacent(s)	Lower adjacent(s)	Median value(s)	Number of outliers
1	The constant is 1	369.049	364.151	366.343	3
2	(16)	468.018	446.098	459.983	3
3	(17)	468.646	446.225	459.6345	2
4	(18)	443.129	394.638	148.49	0
5	(19)	455.38	443.456	448.798	2
6	(20)	461.342	451.524	455.122	3

**Table 3:** Comparison of various indexes under different inertia weights.

Different inertial weight algorithm	Maximum of *V*_best_	Mean value of *V*_ave_	Median value of *V*_ave_	The Euclidean norm of the global error vector	Convergence generation number	The Euclidean norm of the normalized error vector after reaching the convergent generation number
The constant is 1	93.6044%	90.1465%	63.3698%	90.6721	—	—
(16)	94.0279%	93.6873%	93.7169%	8.4488	9	0.0294
(17)	93.9941%	93.2348%	93.6419%	28.2732	30	0.0348
(18)	94.0290%	93.2050%	93.6719%	31.5911	36	0.0320
(19)	94.0044%	93.2996%	93.6618%	27.2316	22	0.0324
(20)	93.9926%	93.2350%	93.6206%	28.1948	27	0.0341
